# The yeast genus *Tardiomyces* gen. nov. with one new species and two new combinations

**DOI:** 10.1007/s15010-024-02229-6

**Published:** 2024-04-04

**Authors:** Bram Spruijtenburg, Bruna Jacomel Favoreto de Souza Lima, Sonia T. Granadillo Tosar, Andrew M. Borman, Cecilie Torp Andersen, Summiya Nizamuddin, Suhail Ahmad, João Nobrega de Almeida Junior, Vânia Aparecida Vicente, Joshua D. Nosanchuk, Jochem B. Buil, Sybren de Hoog, Eelco F. J. Meijer, Jacques F. Meis, Theun de Groot

**Affiliations:** 1 Department of Medical Microbiology, Radboudumc, Nijmegen, The Netherlands; 2 Radboudumc-CWZ Center of Expertise for Mycology, Nijmegen, The Netherlands; 3 Canisius-Wilhelmina Hospital (CWZ)/Dicoon, Nijmegen, The Netherlands; 4 Microbiology, Parasitology and Pathology Post-Graduation Program, Department of Pathology, Federal University of Paraná, Curitiba, Paraná Brazil; 5 UK Health Security Agency National Mycology Reference Laboratory, Southmead Hospital, Bristol, BS10 5NB UK; 6 Medical Research Council Centre for Medical Mycology, University of Exeter, Exeter, EX4 4QD UK; 7 Department of Microbiology, Oslo University Hospital, Rikshospitalet, Oslo, Norway; 8 Section of Microbiology, Shaukat Khanum Memorial Cancer Hospital and Research Centre, Lahore, Pakistan; 9 Department of Microbiology, Faculty of Medicine, Kuwait University, Safat, Kuwait; 10 Laboratório Especial de Micologia, Disciplina de Infectologia, Universidade Federal de São Paulo, São Paulo, Brazil; 11 Bioprocess Engineering and Biotechnology Graduate Program, Federal University of Paraná, Curitiba, Brazil; 12 Microbiological Collections of Paraná Network (CMRP/Taxonline), Department of Basic Pathology, Federal University of Paraná, Curitiba, Brazil; 13 Department of Medicine (Division of Infectious Diseases) and Department of Microbiology and Immunology, Albert Einstein College of Medicine, New York, NY USA; 14 Institute of Translational Research, Cologne Excellence Cluster On Cellular Stress Responses in Aging-Associated Diseases (CECAD) and Excellence Center for Medical Mycology, University of Cologne, Cologne, Germany

**Keywords:** *Tardiomyces depauwii*, *Candida blankii*, Antifungal resistance, Whole-genome sequencing, Misidentification, Characterization, Nomenclature

## Abstract

**Purpose:**

Rare yeasts species are increasingly reported as causative agents of invasive human infection. Proper identification and antifungal therapy are essential to manage these infections. *Candida blankii* is one of these emerging pathogens and is known for its reduced susceptibility to multiple antifungals.

**Methods:**

To obtain more insight into the characteristics of this species, 26 isolates reported as *C. blankii* were investigated using genetic and phenotypical approaches.

**Results:**

Among the 26 isolates, seven recovered either from blood, sputum, urine, or the oral cavity, displayed substantial genetic and some phenotypical differences compared to the other isolates, which were confirmed as *C. blankii*. We consider these seven strains to represent a novel species, *Tardiomyces depauwii*. Phylogenomics assigned *C. blankii, C. digboiensis,* and the novel species in a distinct branch within the order *Dipodascales*, for which the novel genus *Tardiomyces* is erected. The new combinations *Tardiomyces blankii* and *Tardiomyces digboiensis* are introduced. Differences with related, strictly environmental genera *Sugiyamaella, Crinitomyces,* and *Diddensiella* are enumerated. All three *Tardiomyces* species share the rare ability to grow up to 42 °C, display slower growth in nutrient-poor media, and show a reduced susceptibility to azoles and echinocandins. Characteristics of *T. depauwii* include high MIC values with voriconazole and a unique protein pattern.

**Conclusion:**

We propose the novel yeast species *Tardiomyces depauwii* and the transfer of *C. blankii* and *C. digboiensis* to the novel *Tardiomyces* genus.

**Supplementary Information:**

The online version contains supplementary material available at 10.1007/s15010-024-02229-6.

## Introduction

Yeast species are among the primary causative agents of invasive fungal infections. In recent years, invasive infections due to less common species are increasingly reported, partially due to advances in molecular diagnostics and an increase in vulnerable hosts [1–3]. Some of these emerging species pose a threat to public health due to difficulties in identification, limited therapeutic options caused by resistance and the risk of nosocomial outbreaks with undetermined sources of infection [4–7]. Originally described in the organs of a mink in 1968 [8], *Candida blankii* is considered an emerging human pathogenic fungus that has been isolated from varying environments like the surface of apples, flowers, and was the most abundant species in surface coastal marine habitats in Vietnam [9–11]. Environmental *C. blankii* isolates have been used in biotechnological research [12]. The first clinical isolate of *C. blankii* was reported in a cystic fibrosis (CF) patient in 2015 [13]. Since then, *C. blankii* infections were reported worldwide in patients with endocarditis, cystic fibrosis (CF), SARS-CoV-2, and in a preterm neonate [14–18]. Furthermore, *C. blankii* was the causative agent of an outbreak in a neonatal intensive care unit in Delhi, India, which resulted in bloodstream infections in nine neonates over a seven-month period [19]. Additional clinical isolates were retrospectively identified in surveillance studies in Norway and the United Kingdom, albeit in low numbers [20, 21]. A shared characteristic among all investigated strains is the reduced susceptibility against various azoles and echinocandins, making this species difficult to manage. Identification via phenotypic methods appeared challenging, and therefore molecular identification methods are preferred [16].

The closest relative of *C. blankii* is *C. digboiensis*, isolated from sludge-contaminated soil, tar and acid mine drainage in Zambia and India [22–24]. Recently, *C. digboiensis* was reported as the causative agent of candidemia in the United Kingdom [25]. ITS rDNA sequences of both species clustered them in a distinct branch, distant from the CTG clade that includes common *Candida* species like *Candida albicans, Candida tropicalis,* and *Candida parapsilosis* [14].

In the present study, we demonstrate that several European clinical isolates, previously reported as *C. blankii*, were found to harbor genomic and phenotypical differences to isolates curated under the same name, while demonstrating minimal genetic diversity between each other. For the latter cohesive group, we propose a novel yeast species, closely related to *C. blankii*. Given the large phylogenetic distance of both species to *C. albicans,* a model type of the genus *Candida*, we propose the novel genus *Tardiomyces* to accommodate this yeast. With this naming, the reassignment of the previously known *C. blankii* and *C. digboiensis* to the genus *Tardiomyces,* thus, is necessary.

## Materials and methods

### Isolate culture and DNA extraction

A total of 26 isolates initially reported as *C. blankii* were cultured for two days on Sabouraud dextrose agar (SDA) (Oxoid, Hampshire, United Kingdom) at 30 °C after being taken from storage at −80 °C (Table S1). *C. albicans* American Type Culture Collection (ATCC) 10231*, C. tropicalis* ATCC 750^T^*, C. parapsilosis* ATCC 22019^T^*, Pichia kudriavzevii* (*Candida krusei*) ATCC 6258^T^*,* and *Nakaseomyces glabratus* (*Candida glabrata*) ATCC 90030 were used in each experiment as controls. For each isolate, a colony was randomly selected and resuspended in 700 µL MagNA Pure Bacteria lysis buffer and MagNA Lyser green beads and mechanically lysed for 30 s at 6500 rpm with the MagNA Lyser system (All Roche Diagnostics GmbH, Mannheim, Germany). DNA was extracted and purified with the MagNA Pure 96 instrument with the MagNA Pure DNA and Viral Small Volume Kit (Roche Diagnostics), following the manufacturer’s instruction.

### Identification methods

Isolates were analyzed by matrix-assisted laser desorption ionization-time of flight (MALDI-TOF) mass spectrometry (MS) using a Bruker MALDI Biotyper system as described [26]. Biochemical profile analysis with a VITEK 2XL (BioMérieux, Marcy l’Etoile, France) was done according to manufacturer’s instruction using VITEK 2 YST ID card and VITEK 2XL identification software v9.02 and were analyzed in triplicates. For internal transcribed spacers (ITS) sequencing, ITS-1 and ITS-4 primers were used as described [27]. Control sequences were extracted from the National Center for Biotechnology Information (NCBI) nucleotide database and alignment was generated using MAFFT v7 [28], and the phylogenetic tree was built with IQ-TREE web server with default parameters [29] (Table S2). Visualization and edition were made using iTOL v6 [30]. ITS sequences generated in the current study were deposited to the NCBI Genbank database (Accession numbers: OR479767–OR479792).

### Whole-genome sequencing (WGS)

For WGS, DNA was extracted as described above and samples were subsequently treated with RNase A (Merck KGaA, Darmstadt, Germany) at a final concentration of 5 µg/µL for one hour at room temperature. DNA was afterward extracted and purified again as before and then measured with a Qubit 3.0 Fluorometer (Thermo Fisher Scientific, Waltham, MA, USA) using the double-stranded DNA (dsDNA) high-sensitivity option. For Illumina sequencing, genomic libraries were prepared and sequenced with the Illumina NovaSeq 6000 platform (Illumina, San Diego, CA, USA) with 2-by-150 bp paired-end-read mode at Eurofins Genomics (Ebersberg, Germany). For Oxford Nanopore sequencing, libraries were prepared using the ONT rapid sequencing kit and were sequenced using a GridION (Oxford Nanopore Technologies [ONT], Oxford, UK) instrument. Raw read data generated in this study have been submitted to the NCBI Sequence Read Archive (BioProject ID: PRJNA1009266).

### Assembly and annotation

Generated Nanopore and Illumina reads were uploaded to the Galaxy platform and were assessed with FastQC [31]. De novo assembly was performed on Nanopore reads with Flye v2.9.1 using the Nanopore raw mode [32]. Illumina reads of the same strain were aligned to the generated assembly using BWA-MEM v0.7.17, PCR duplicates were removed with RmDup, local realignment was performed using BamLeftAlign and unpaired reads were removed with BAM filter [33]. The draft assembly was improved using the generated BAM file and Pilon v.1.20.1 [34]. Gene prediction and annotation were performed using Funannotate pipeline v1.8.15 [35], including soft masking with RepeatMasker and RepeatModeler tools, ab initio training using Augustus, Snap, glimmerHMM, codingQuary, and GenMark. Functional annotation was assigned by similarity using Pfam, MEROPS, CAZy, eggNOG, InterProScan, Antismash, SignalP, and Swissprot databases by BLASTP or HMMER3 searches using Funannotate default parameters. Genome annotations are available online (Supplementary table). Candidate heterozygous positions predicted by Pilon v1.20.1 were examined using JBrowse v1.16.11 [36]. For de novo assembly based on Illumina reads, SPAdes v3.15.4 with default settings was used except for the assembly and error correction operation mode and paired-end: individual datasets reads options [37]. Genome assembly qualities was assessed with Quast v5.2.0 [37]. The generated assembly was deposited to the NCBI Assembly database (BioProject ID: PRJNA1009997).

### Phylogenomic analysis and comparative genomics

Orthology of 39 species of *Saccharomycotina* subphylum was accessed using OrthoFinder v2.5.4 [38], with default parameters to identify orthologous gene groups and Multiple Sequence Alignment option to infer a rooted species tree (Table S3). *Schizosaccharomyces pombe* and *Cryptococcus neoformans* were used as outgroup species. The generated tree was visualized and edited using iTol v6 [30]. Orthologous gene clusters of *Tardiomyces depauwii, T. blankii, T. digboiensis,* and two closely related species *Crinitomyces ghanaensis* and *Sugiyamaella lignohabitans* were compared using OrthoVenn2 using an e-value cutoff of 1 × 10^–5^ and inflation values of 1.5 [39].

SNPs in *T. depauwii* isolates were called using Freebayes v.1.3.6 as previously described using a validated pipeline [40]. In short, SNPs in the resulting VCF file with a read depth (DP) of < 15, a quality of < 100 and an allele frequency between 0.15 and 0.90 × DP were removed. Phylogenetic analysis and visualization were performed using VCF2PopTree, MEGA11 v11.0.10 and iTOL v6 as previously described [41].

Bayesian phylogenies were generated with BEAST v.1.10.4 [42]. The filtered VCF file was converted to FASTA alignments with vcf2phylip v2.8 and was subsequently converted to an xml file with BEAUti v1.10.4 [43]. Random nucleotides totaling the *T. depauwii* genomic size minus the number of positions on the VCF file were inserted in the generated xml file. Tip dates were set to the year of collection. The GTR model with gamma variation among sites was used as the substitution model with a strict molecular clock. A coalescent exponential growth model was selected as the Tree Prior and the length of chain was set to 50,000,000. The generated log file was inspected with Tracer v1.7.2, annotation was performed with TreeAnnotator v1.10.4 with the burnin (as states) set to 5,000,000 and subsequently visualized with FigTree v1.4.4 [44].

### Phenotypic characterization

Micro- and macroscopic morphology were determined after 48 h of incubation on yeast peptone dextrose (YPD) plates (Thermo Fisher Scientific) and Candi*Select* Agar (Bio-Rad Laboratories, Inc., Hercules, CA, USA) at 30 °C. Physiological characterization of temperature effects, osmotolerance, NaCl stress, and urease hydrolysis on growth was performed using standard procedures described in [45]. API 20 C AUX (BioMérieux), strips were used as suggested by the manufacturer and incubated at 30 °C for 72 h. Assimilation patterns were read after 72 h of incubation. For measuring growth rates of *T. depauwii, T. blankii, T. digboiensis,* and common yeast species (*C. albicans, C. tropicalis, C. parapsilosis, P. kudriavzevii, N. glabratus)*, cell suspensions were diluted in water to a final concentration of 1–5 × 10^3^ CFU/mL using a Genesys 20 Spectrometer (Thermo Fisher Scientific). Cultures were incubated at 35 °C and growth was quantitated by measuring optical density (OD) at 450 nm, using an 800 TS Microplate Reader (BioTek, Winooski, VA, USA). Isolates were incubated in YPD broth (Thermo Fisher Scientific), SAB (Oxoid) and RPMI 1640 medium with 0.2% and also 2% glucose (Thermo Fisher Scientific). The experiment was replicated eight times and averages with standard error of the mean were calculated using Prism v8.4.3 (Boston, MA, USA).

### Antifungal susceptibility testing (AFST)

In vitro AFST in accordance with the Clinical & Laboratory Standards Institute (CLSI) M27 standard was performed against eight common antifungals [46]. In short, isolates were diluted to obtain a final concentration of 1–5 × 10^3^ CFU-mL in RPMI 1640 medium (Thermo Fisher Scientific). Microtiter plates were incubated at 35 °C and visually read after 24 and 48 h as the first reading showed inadequate growth. Minimal inhibitory concentration (MIC) values were determined as the lowest antifungal concentration with a 50% growth reduction when compared to the growth control, except amphotericin B with a 100% growth reduction. Tested concentrations for all drugs ranged between 0.016 and 16 µg/mL, except for fluconazole with a range of 0.063–64 µg/mL. The Sensititre YeastOne (SYO) YO10 (Thermo Fisher Scientific) AFST was performed following manufacturer’s recommendations. Microtiter plates were incubated at 35 °C for 48 h, as incubation after 24 h did not yield sufficient growth, and SYO colorimetric MICs were recorded as the first well showing a distinct color change as compared to the positive control. AFST by E-test for amphotericin B was performed for all *T. depauwii* isolates. Inoculum suspensions of 0.5 McFarland were inoculated on the entire surface of the RPMI plate with a sterile cotton swab. E-test strips (Liofilchem, Roseto degli Abruzzi, Italy) were placed on the center of the RPMI 1640 plate and incubated at 35 °C for 72 h. The MIC was determined from the inhibition ellipse that intersected with the scale of the strip after 48 and 72 h as 24 h of incubation showed not enough growth.

## Results

### Isolate identification

**Fig. 1:**
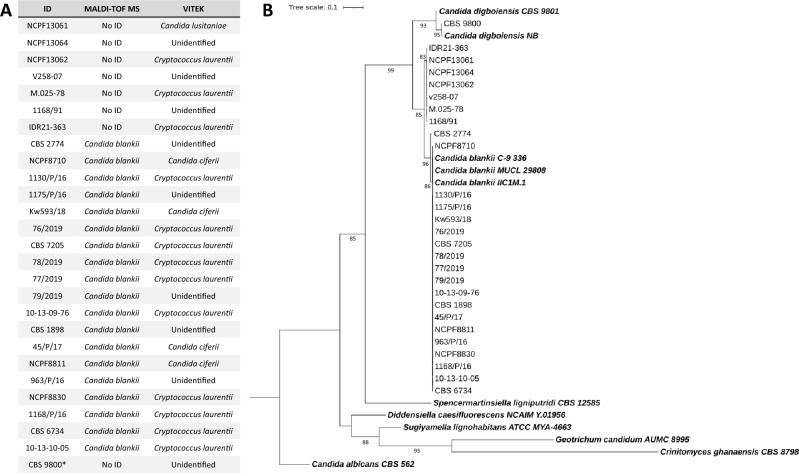
Identification results according to ITS sequencing, MALDI-TOF MS and VITEK. **A** MALDI-TOF MS and VITEK analysis were used to identify the isolates. *, CBS 9800 is *C. digboiensis* and included as control. **B** Phylogenetic tree of species from the *Dipodascales* order based on ITS sequences. The tree is constructed with maximum likelihood implemented in IQ-TREE web server using TPM3u + F + G4 model. Ultrafast bootstrap was used to 1000 resampled data and values > 80 are shown. Strains retrieved from NCBI are indicated in bold. *Candida albicans* was taken as an outgroup

All isolates, reported previously as *C. blankii*, were investigated by means of MALDI-TOF MS, ITS sequencing and the VITEK 2XL system. We also included a single *C. digboiensis* strain (type strain CBS 9800), the most related species to *C. blankii*. Out of 26 isolates, 19 were identified as *C. blankii* by MALDI-TOF MS, with score values of ≥ 1.7, and a 100% ITS sequence similarity match to multiple annotated *C. blankii* strains submitted to the NCBI Nucleotide database, also confirmed by phylogenetic analysis (Fig. 1A and B). The remaining seven strains could not be identified with MALDI-TOF MS, and ITS analysis displayed a difference of 21 substitutions and two indels from other *C. blankii.* For CBS 9800, identification using MALDI-TOF MS was also not possible. VITEK 2XL results of all isolates yielded either *Cryptococcus laurentii* (*n* = 13), *Candida ciferrii* (*n* = 4)*, Clavispora (Candida) lusitaniae* (*n* = 1)*,* or unidentified species (*n* = 9).

### Phylogenetic placement

**Fig. 2:**
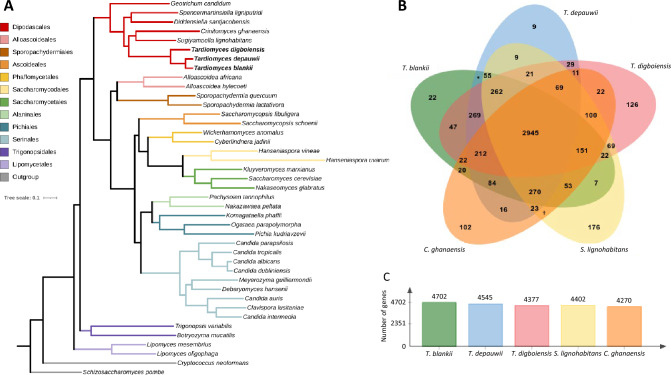
Phylogenetic analysis based on ortholog groups from *Saccharomycotina* including the *Tardiomyces* genus. **A** Species tree based on proteomes from *Saccharomycotina* built from 2843 orthologous groups of genes. *Tardiomyces* spp. are highlighted. *Cryptococcus neoformans* and *Schizosaccharomyces pombe* were taken as outgroups. **B** Venn diagram of *T. depauwii, T. blankii, T. digboiensis, C. ghanaensis* and *S. lignohabitans* showing the number of shared and unique gene clusters for each species. **C** Overview of the total number of genes for each species. *3205 shared genes. †83 shared genes

From the seven strains that were not identified as *C. blankii* with MALDI-TOF, strain NCPF13064 was used to generate a high quality de novo assembly using Nanopore reads with Illumina read correction. This assembly generated nine contigs, with lengths ranging from 12 Kbp to 3.82 Mbp, yielding a total length of 13.47 Mbp (N_50_ = 3,623,071 bp, GC% = 57.69%). Genome annotation yielded 4867 protein-coding genes. Inspection of potential heterozygous positions according to aligned Illumina reads did not reveal such positions, indicating a haploid genome. Using this strain and type strains of *C. blankii*, *C. digboiensis*, 36 other *Saccharomycotina* genomes and two outgroups, comparative genomics was used to construct a phylogenomic tree based on ortholog groups, which showed a congruent topology in *Saccharomycotina*, grouped within the order *Dipodascales*, indicating a close relation between strain NCPF13064 and *C. blankii* and a common ancestor together with *C. digboiensis* (Fig. 2A). Strain NCPF13064 was differentiated from *C. blankii*, at a similar distance as found between the sister species *C. albicans* and *C. dubliniensis*, demonstrating that this strain together with the other five related isolates constitute a different, previously unrecognized species. Bases on this, we propose to name this novel species as *Tardiomyces depauwii.* Isolates of this species were isolated in Europe (Norway, the United Kingdom and the Netherlands) and the United States (New York), between 1991 and 2021 (Fig. S1). Furthermore, we propose the reassignment of the related *C. blankii* and *C. digboiensis* to the genus *Tardiomyces*, as they are grouped with *T. depauwii* and differ from the other described *Candida* species. With comparative genomic analysis, *T. depauwii* exhibited nine unique genes in comparison to the other species. For *T. blankii* and *T. digboiensis,* this number was 22 and 126 respectively (Fig. 2B). The number of genes for species in the genus *Tardiomyces* ranged between 4377 and 4702 (Fig. 2C).

**Fig. 3:**
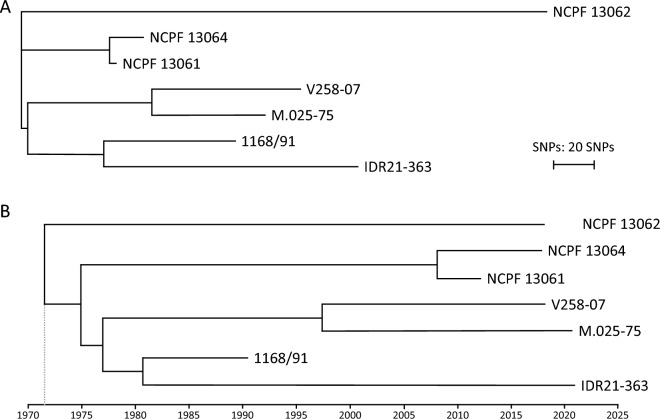
WGS SNP and molecular clock analysis on all *T. depauwii* strains. **A** WGS SNP analysis, the tree was generated with MEGA11 v11.10.11 using the neighbor-joining tree method. **B** Molecular clock analysis with estimated dates of the most recent common ancestor. The tree was generated with BEAST v.1.10.4 using a strict clock with a coalescent model

WGS SNP analysis was performed on all seven *T. depauwii* isolates. SNP differences ranged from 23 to 459 SNPs between strains on a genome of approximately 13.5 Mbp (Fig. 3A). Divergence times between isolates were estimated with Bayesian inference utilizing the GTR substitution model with a coalescent exponential growth. Using Tracer, ESS values were sufficient for all parameters. The estimated time to the most recent common ancestor (TMRCA) for these *T. depauwii* isolates was as recent as around 1971 (Fig. 3B). Finally, using this pipeline it was not possible to map most reads of the *T. depauwii* isolates on the *C. blankii* reference genome NCPF13064.

### Phenotypic characterization

**Fig. 4:**
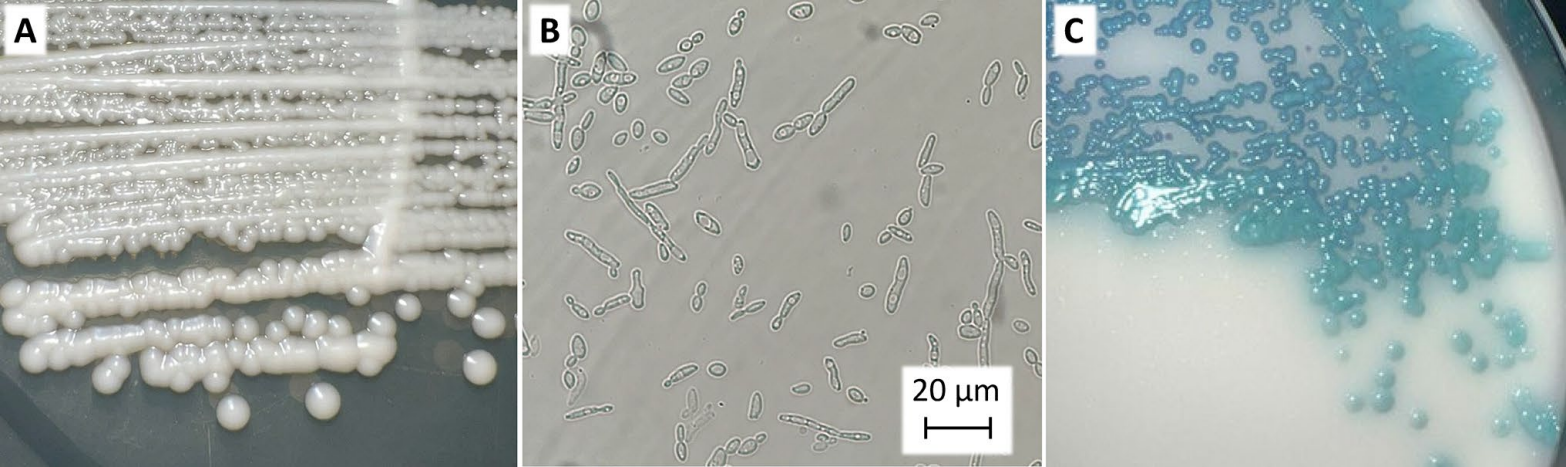
*T. depauwii* morphology. **A** Macroscopic morphology of *T. depauwii* after 48 h on YPD agar. **B** Microscopic morphology of *T. depauwii* after 48 h in YPD broth. **C** Morphology of *T. depauwii* on Candi*Select* Agar

All seven *T. depauwii* strains formed smooth creamy white colored colonies after incubation for 48 h on YPD agar (Fig. 4A). Isolates propagated by means of budding cells, which were ellipsoidal to subspherical, 3–5 µm by 3–7 µm or via the formation of pseudohyphae (Fig. 4B). On Candi*Select* Agar, colonies appeared light to dark blue (Fig. 4C). The six strains did not show any morphological differences between each other. Physiological characteristics regarding fermentation and assimilation of carbon sources, osmo- and saline tolerance, urease hydrolysis, and thermotolerance, were examined for all *T. depauwii, T. blankii* and *T. digboiensis* strains (Table 1). The fermentation and assimilation of carbon sources were highly similar between these species with only few differences. No phenotypic intra-species variation among *T. depauwii* strains was observed, while for the *T. blankii* strains five substrates were variably assimilated (Table S4). Phenotypical results for 16 substrates included in the API 20 C AUX confirmed the results mentioned above (Table S5). While *T. blankii* was able to grow in up to 60% glucose and 10% NaCl, *T. depauwii* and *T. digboiensis* did not show growth at 50% glucose and grew at a maximum saline concentration of 7.5%. All three investigated *Tardiomyces* species displayed growth up to 42 °C.

**Table 1:** Phenotypic characteristics of *Tardiomyces depauwii, T. blankii* and *T. digboiensis*

Characteristic	*T. depauwii* (*n* = 7)	*T. blankii* (*n* = 19)	*T. digboiensis* (*n* = 1)
*Fermentation of carbon sources*			
Galactose	+	+	+
Maltose	+	+	+
Sucrose	+	+	+
Raffinose	−	−	+
Trehalose	+	+	−
*Assimilation of carbon sources*			
Arbutin	+	+	−
Amygdalin	−	−	−
Cellobiose	+	+^a^	+
Citrate	+	−^a^	−
D-mannose	+	+	+
D-turanose	+	+	+
D-xylose	+	+	+
DL-lactate	−	−	+
Erythritol	+	+	+
Galactose	+	+^a^	+
Gentobiose	+	+	+
Glucuronate	+	+	+
L-arabinose	+	+	+
L-glutamate	+	+	+
L-rhamnose	+	+	+
L-sorbose	+	+	+
Melezitose	−	−	+
Melibiose	−	−	+
Nitrate	−	−	−
Methyl-α-D-glucoside	−	+^a^	+
N-acetyl-glucosamine	+	+^a^	+
Raffinose	−	−	+
Sorbitol	+	+	−
Xylitol	+	+	+
2-keto-D-gluconate	+	+	−
*Other tests*			
Osmotolerance (50% w/w)	−	+	−
Osmotolerance (60% w/w)	−	+	−
NaCl tolerance (5% w/v)	+	+	+
NaCl tolerance (7.5% w/v)	+	+	+
NaCl tolerance (10% w/v)	−	+	−
Urease hydrolysis	−	−	−
*Growth on SAB agar at temperature*			
20 °C	+	+	+
30 °C	+	+	+
35 °C	+	+	+
42 °C	+	+	+
47 °C	−	−	−

Scoring system according to Kurtzman et al. [45], + : positive test, − : negative test.

^a^Assimilation results were variable between strains

### Growth profile

**Fig. 5:**
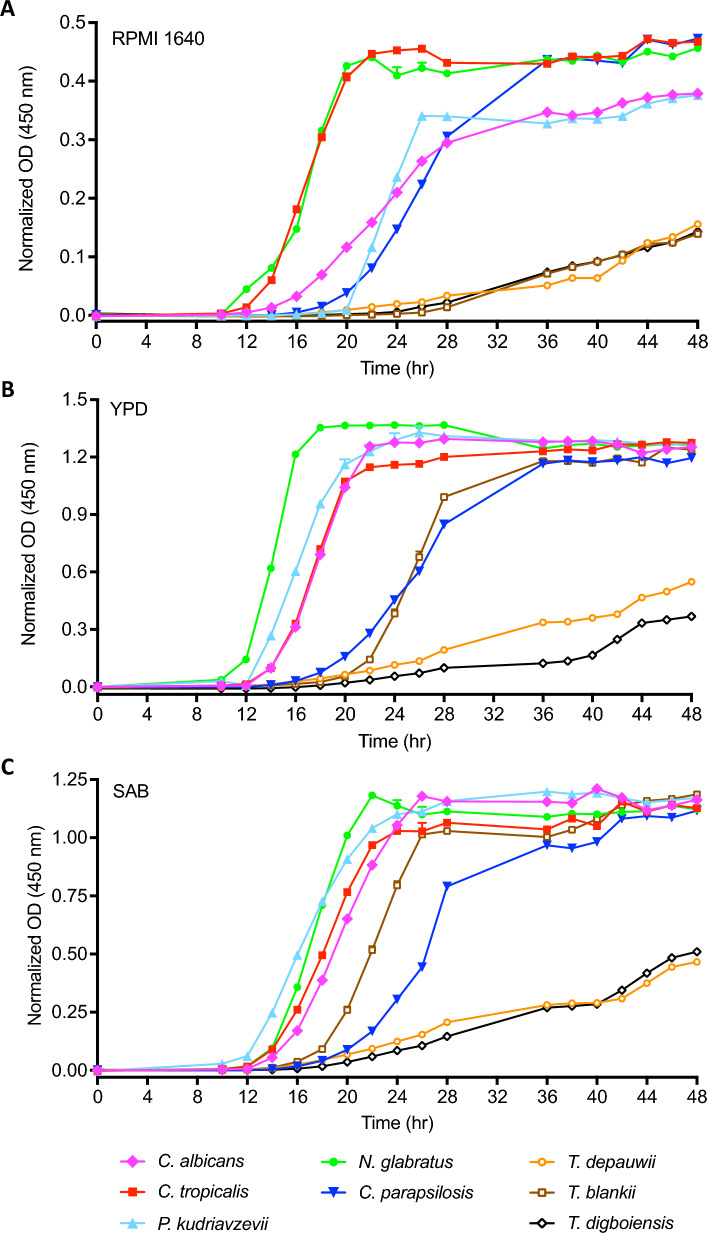
Growth curves of *Tardiomyces* and common yeast species cultured in RPMI 1640 (0.2% glucose) medium (**A**), YPD (**B**) and SAB (**C**) broth at 35 °C. One strain of each species was grown in eightfold and growth is expressed in average normalized OD values

Growth rates of randomly selected *T. depauwii* (*n* = 3), *T. blankii* (*n* = 4),* T. digboiensis* (*n* = 1) and five other common human pathogenic yeast species (*C. albicans, C. tropicalis, C. parapsilosis, P. kudriavzevii* and *N. glabratus*) were investigated in RPMI 1640 medium (0.2% glucose), YPD broth and SAB broth. For RPMI 1640 with 0.2% glucose, all common yeast species reached the stationary phase around 24 h of incubation at 35 °C, while all isolates from the *Tardiomyces* genus exhibited a much slower growth rate, showing continuous growth till 48 h, with no indication of having reached the stationary phase (Fig. 5A). In YPD broth and SAB broth, all *T. depauwii* and *T. digboiensis* strains showed a greatly reduced growth rate when compared to the control strains, displaying continuous growth at 48 h, while most control strains reached the stationary phase around 24 h of incubation (Fig. 5B, C). Notably, the three *T. blankii* strains grew with the same rate as the common yeast species and one showed similar growth rates as *T. depauwii* and *T. digboiensis* (Fig. S2)*.* To determine whether the higher glucose content of these media was involved, we also grew all strains in RPMI 1640 medium with 2% glucose (Fig. S3). Also, in this medium the same *T. blankii* strains demonstrated an elevated growth rate, as compared to the *T. depauwii* and *T. digboiensis* strains, however, they did not reach a growth rate as found for the common yeast strains (Fig. S2).

### Antifungal susceptibility

In vitro AFST according to CLSI microbroth dilution was performed against eight common antifungals on all *T. depauwii isolates* (*n* = 7) and on *T. blankii* (*n* = 4) and *T. digboiensis* (*n* = 1) isolates as used before in the growth rate experiment. Due to minimal growth at 24 h of incubation, MICs values were read after 48 h of incubation. Notably, the *T. depauwii* strains demonstrated elevated fluconazole and voriconazole MICs of ≥ 64 µg/mL and 2–8 µg/mL, respectively (Table 2). For *T. blankii,* fluconazole MICs ranged from 8 to 32 µg/mL in the four tested strains, while the *T. digboiensis* isolate showed a MIC of 4 µg/mL. Anidulafungin and micafungin MICs were elevated for nearly all *Tardiomyces* strains, ranging mostly between 0.125 and 8 µg/mL. Overall, anidulafungin MICs were twofold dilutions higher than micafungin MICs for *T. depauwii* and *T. blankii*. Amphotericin B MICs were either 0.25 or 0.5 µg/mL for all tested *Tardiomyces* isolates. MIC values according to SYO were similar, confirming the elevated MICs for most azoles and echinocandins of all *Tardiomyces* strains and the higher MICs of anidulafungin when compared to micafungin. Finally, SYO demonstrated that flucytosine MICs were low, with MICs being ≤ 0.12 µg/mL (Table S6). Amphotericin B MICs for *T. depauwii* strains using E-tests were read after 48 and 72 h and ranged between 0.38 and 1 µg/mL, and 0.5 and 1 µg/mL, respectively (Table S7).

**Table 2:** In vitro AFST minimal inhibitory concentrations for *Tardiomyces depauwii* (*n* = 7), *T. blankii* (*n* = 4) and *T. digboiensis* (*n* = 1) according to CLSI M27-S4 standard after 48 h incubation. Minimal inhibitory concentrations in µg/mL

Species	ID	AMB MIC	FLC MIC	ITR MIC	VOR MIC	POS MIC	ISA MIC	AFG MIC	MFG MIC
*T. depauwii*	1168/91	0.25	≥ 64	0.25	4	0.5	0.5	4	0.25
	M.025-78	0.5	≥ 64	0.25	8	0.25	0.25	4	1
	V258-07	0.5	≥ 64	0.25	8	0.5	1	0.5	0.125
	NCPF13061	0.25	≥ 64	0.25	4	0.25	0.125	2	0.25
	NCPF13062	025	≥ 64	0.25	2	0.5	0.5	4	0.5
	NCPF13064	0.5	≥ 64	0.5	8	0.5	1	1	0.125
	IDR21-363	0.25	≥ 64	0.5	8	1	1	2	0.25
*T. blankii*	79/2019	0.25	32	0.5	1	0.5	0.25	1	0.063
	Kw593/18	0.25	16	0.5	0.5	0.5	0.125	4	8
	1130/P/16	0.5	32	2	2	2	1	4	0.25
	CBS7205	0.25	8	0.031	0.125	0.125	0.031	0.5	0.063
*T. digboiensis*	CBS9800	0.5	4	0.031	0.125	0.031	0.063	2	2

*MIC* minimal inhibitory concentration, *AMB* amphotericin B, *FLC* fluconazole, *ITR* itraconazole, *VOR* voriconazole, *POS* posaconazole, *ISA* isavuconazole, *AFG* anidulafungin, *MFG* micafungin

## Discussion

In the present study, we described a novel yeast species and proposed the reassignment of *C. blankii* and *C. digboiensis* to a novel genus *Tardiomyces*. Genotypic analysis of the seven clinical *T. depauwii* isolates revealed an extremely low genetic diversity, despite originating from different countries during a 32-year period, and clear delineation from the related *T. blankii (C. blankii)* and *T. digboiensis (C. digboiensis)*. The genus *Tardiomyces* is characterized by an overall low growth rate in comparison to common pathogenic yeast species, elevated MICs against azoles and echinocandins, especially against fluconazole, voriconazole and anidulafungin in most of the strains, and growth up to 42 °C.

### Identification and genetics of *Tardiomyces*

Recent advances in molecular taxonomy have facilitated the increase of reported rare yeasts, and the description of novel species and genera [47]. Molecular identification methods like ITS sequencing or WGS analysis are considered as gold standard due to their high discriminatory power when compared to biochemical methods [48]. With ITS sequencing, genetic variations were observed between *T. depauwii* and *T. blankii*, with a number of mismatches similar to those of other yeast sibling species [49]. While genetic variation in barcoding genes including ITS is minimal between some species [47], ITS sequencing showed a clear distinction between *T. depauwii* and the related *T. blankii* and *T. digboiensis*, which was confirmed by comparative genomics. Furthermore, all three *Tardiomyces* species differed in the total number of genes, each exhibiting unique genes that were not found in related species. In addition to genomic differences, phenotypical variation between the three species was observed in enzymatic activity, saline- and osmotolerance. Phenotypical tests, besides the current ITS or WGS analyses, can therefore be used to differentiate *T. depauwii* from related species in routine clinical settings. It should be noted that few *T. depauwii* isolates were tested. With the identification of more isolates, the phenotypical variation will likely increase.

In the last decade, the nomenclature of medically important fungi underwent significant changes due to phylogenetic reassignment of genera, leading to name changes of important species including *C. glabrata, C. krusei, Candida kefyr,* and *Candida guilliermondii,* renamed as *Nakaseomyces glabratus, Pichia kudriavzevii, Kluyveromyces marxianus,* and *Meyerozyma guilliermondii*, respectively [50]. Originally, phenotypic methods were used to characterize yeasts [51]. With phylogenetics, species previously assigned to the genus *Candida* are often polyphyletic and exhibit widely different characteristics, which do not fit the definition of a genus [52]. The allocation of *T. depauwii, T. blankie,* and *T. digboiensis* in a novel genus is more in line with the novel approach [53].

When WGS SNP analysis was applied to the *T. depauwii* strains, an extremely low genetic diversity of less than 500 SNPs between strains was observed. This, in addition to the estimated TMRCA at 1971, indicates a recent introduction into the human population. The isolation of strains from the oral cavity, sputum and urine suggests that *T. depauwii* can colonize humans, while its extremely low genetic diversity in combination with its epidemiology suggests a single introduction into the human population and subsequent spread via human-to-human transmission.

### Physiological properties

A shared characteristic of species within the *Tardiomyces* genus is their ability to grow at up to 42 °C, which is rarely observed for other fungi that mostly thrive in the temperature range of 12–30 °C [54]. The genetically related genera *Sugiyamaella* and *Crinitomyces* do not grow at 42 °C [55, 56]. The *Tardiomyces* genus is, therefore, thermotolerant to mammalian temperatures despite all *T. depauwii* isolates originating from countries with temperate climates. A mammalian reservoir of the latter species could explain such thermotolerance. It is however not likely that *T. depauwii* is restricted to such high-temperature reservoirs as *T. blankii* and *T. digboiensis* strains have also been isolated from varying environments with substantially lower temperatures.

Relatively slow growth was observed for *T. depauwii, T. digboiensis,* and *T. blankii* in RPMI 1640 medium with 0.2% glucose when compared to other common yeast species, including *C. parapsilosis* that is known for its low growth rate [57]. Interestingly, when the experiment was replicated in RPMI 1640 medium with 2% glucose, some *T. blankii* strains exhibited an elevated growth rate when compared to RPMI 1640 medium with 0.2%. Thus, a lack of glucose is hampering the growth of some *T. blankii* strains while others seem to be indifferent to its availability. However, the elevated growth rate of these *T. blankii* strains in this medium was still lower as compared to YPD and SAB broth, indicating multiple nutrients influence the growth rate of these strains [57]. Independently from the available nutrients, *T. depauwii* and *T. digboiensis* seem to have an intrinsic low growth speed. As a limitation of this study, it must be noted that genetic diversity of *T. depauwii* was very low and only a single *T. digboiensis* isolate was included. Finally, we found that all *Tardiomyces* strains exhibit the ability to assimilate a wide range of substrates, which was recently also found for *T. blankii* [58].

### Antifungal susceptibility

Due to the slow growth of different *Tardiomyces* strains, CLSI, EUCAST, and SYO AFST MICs should ideally be read at 48 h, at which antifungals are still potent and MICs can be interpreted reliably [59, 60]. Incubation for longer periods might induce a trailing effect, resulting in unreliable MICs, and is not recommended. Despite a lack of clinical breakpoints and epidemiological cutoff values, *T. depauwii* can be considered as resistant to fluconazole and voriconazole as high MICs were observed [61]. Susceptibility testing of genetically different strains is required to determine whether this resistance is intrinsic or acquired from a medical or environmental setting [62]. *T. blankii* and *T. digboiensis* exhibited elevated MICs for azoles and echinocandins as well, making this likely a worrying genus-wide characteristic.

Despite the apparent low prevalence of *T. depauwii* and *T. blankii* invasive infections, the elevated MICs make accurate species identification crucial for effective clinical management. For invasive candidiasis, echinocandins are considered the empirical drug of choice [63]. There are currently no treatment recommendations for this genus, but MICs of micafungin and caspofungin were more favorable than anidulafungin. In one case, echinocandin monotherapy had a favorable outcome [17]. Subsequent step-down azole therapy can be evaluated based on AFST, but may be unfavorable given the high MIC values. Based on MICs reported here, liposomal amphotericin B might represent an appropriate intravenous treatment alternative [64]. A discrepancy was identified between gold-standard AFST (CLSI M27-S4) and SYO, where MICs were elevated using the SYO method. An overestimation of amphotericin B MICs by SYO has also been described for *C. auris* [65], which can be negated by determining growth inhibition MIC endpoints regardless of the color, as was employed here. A third AFST method using E-test demonstrated MICs after 48 and 72 h were higher than those obtained with regular CLSI AFST but lower than the SYO MICs. The large differences between the echinocandin MICs for *C. blankii*, e.g., anidulafungin versus micafungin and caspofungin, are remarkable as they have the same target. To our knowledge there are no other species in which such differences have been reported.

In summary, we have described the novel yeast species *T. depauwii* and propose reassignment of the related *C. blankii* and *C. digboiensis* to the novel *Tardiomyces* genus according to phylogenomic analyses. Shared characteristics of this novel genus are elevated MICs against azoles and some echinocandins, growth up to 42 °C and an overall relative slow growth rate. All characterized *T. depauwii* strains were of clinical origin with high MICs against fluconazole, voriconazole and anidulafungin, making this a clinically challenging species.

## Taxonomy

### *Tardiomyces* B. Spruijtenburg, J.F. Meis & T. de Groot gen. nov.

*Tardiomyces* (tar.dio.my.ces, from *tardio,* adjective meaning slow and *myces,* fungus).

Growth is via multilateral budding of small cells and the formation of pseudohyphae. Species display growth up to 42 °C under standard conditions and generally display a relatively slow growth rate in YPD broth, SAB broth, and RPMI 1640 medium. Fermentation is absent. D-mannose, D-turanose, D-xylose, erythritol, gentobiose, glucoronate, L-arabinose, L-glutamate, L-rhamnose, L-sorbose, xylitol are assimilated and amygladin is not assimilated for all species. Nitrate is not assimilated.

Type species: *Tardiomyces depauwii*

### *T. depauwii *B. Spruijtenburg, J.F. Meis & T. de Groot* sp. nov.*

*Tardiomyces depauwii* (de.pauw.ii, in honor of emeritus prof. Ben de Pauw for his noteworthy contributions to the advancements of clinical mycology).

MycoBank number: 850439. Holotype: CBS 18495, isolated from human blood in Stoke, United Kingdom, preserved in a metabolically inactive state at the CBS culture collection hosted at the Westerdijk Fungal Biodiversity Institute, Utrecht, the Netherlands. Living strain ex-type CBS 18495 = NCPF 13064.

On YPD agar after seven days of incubation at 30 °C, colonies are smooth creamy white and soft (Fig. 4A**).** In YPD broth, budding cells occur singly or in pairs, are ellipsoidal to subspherical, 3–5 µm by 3–7 µm or display formation of pseudohyphae (Fig. 4B). Pseudohyphae bearing branched chains of ovoid cells. Sporulation is not observed in pure cultures on YPD and SAB agar at 30 °C up to ten weeks. Phenotypic tests and growth characteristics are summarized in Table 2. Elevated MICs against multiple azoles and echinocandins were observed according to CLSI microbroth dilution (Table 2).

### *Tardiomyces blankii* (H.R. Buckley & N. van Uden) B. Spruijtenburg, J.F. Meis & T. de Groot comb. nov.

Basionym: *Candida blankii* H.R. Buckley & N. van Uden. *Mycopathologia et mycologia applicata* **36**(3):257–266 (1968).

Type strain: CBS 1898, isolated from mink organs (Table S1).

### *Tardiomyces digboiensis* (G.S. Prasad, Marilraj, Sood & Lal) B. Spruijtenburg, J.F. Meis & T. de Groot comb. nov.

Basionym: *Candida digboiensis* G.S. Prasad, Marilraj, Sood & Lal. *International journal of systematic and evolutionary microbiology* **55**(2):967–972 (2005).

Type strain: CBS 9800, isolated from acid tar sludge-contaminated soil **(**Table S1).

## Supplementary Information

Below is the link to the electronic supplementary material.

Supplementary file1 Figure S1 Geographic map depicting *T. depauwii* isolate overview including clinical information. Figure adapted from //commons.wikimedia.org/wiki/File:North_Sea_location_map.svg and https://en.m.wikipedia.org/wiki/File:USA_Northeastern.png (PDF 216 KB)

Supplementary file2 Figure S2 Growth curves of *T. depauwii* (n = 3), *T. blankii* (n = 4) and *T. digboiensis* (n = 1) cultured in RPMI 1640 (0.2% glucose) medium (A), YPD (B) and SAB (C) broth at 35 °C. Strains were grown in eightfold and growth is expressed in average normalized OD values (PDF 129 KB)

Supplementary file3 Figure S3 Growth curves of *Tardiomyces* and common yeast species cultured in RPMI 1640 (2% glucose) medium at 35 °C. On top (A), one strain of each *Tardiomyces* species is displayed in addition to controls, and on bottom (B) solely *Tardiomyces* strains are shown. Strains were grown in eightfold and growth is expressed in average normalized OD values (PDF 75 KB)

Supplementary file4 (DOCX 27 KB)

## Data Availability

No datasets were generated or analysed during the current study.
