# Supplementary figures and images for: The yeast genus Tardiomyces gen. nov. with one new species and two new combinations
Source: Infection. 2024 Apr 4;52(5):1799–812. doi: 10.1007/s15010-024-02229-6 (PMC11499460; doi:10.1007/s15010-024-02229-6)

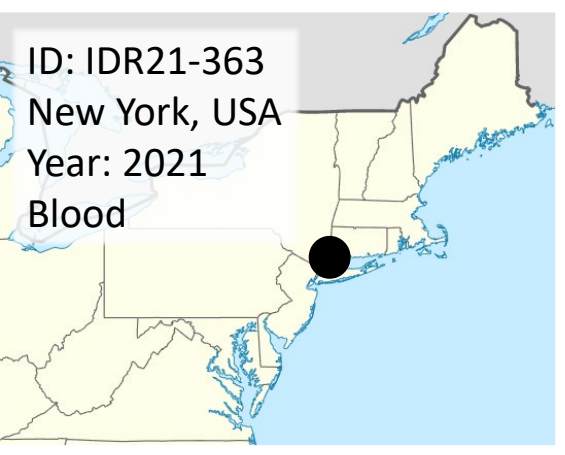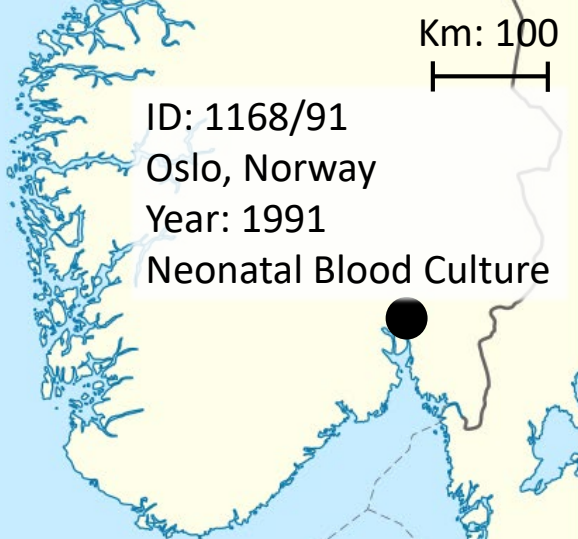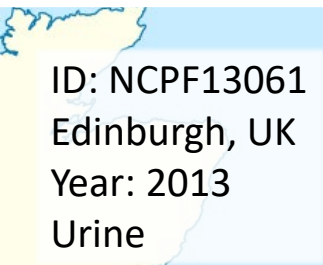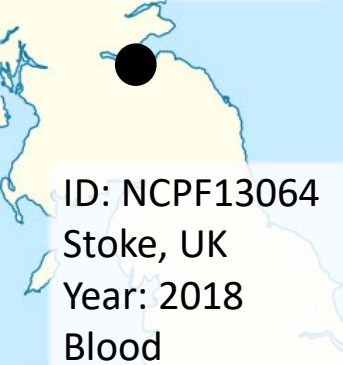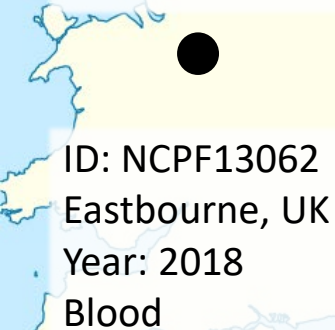

*North Sea*

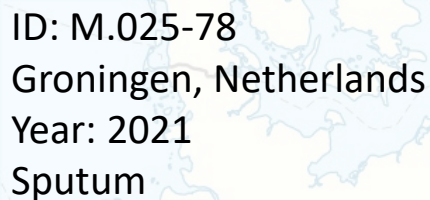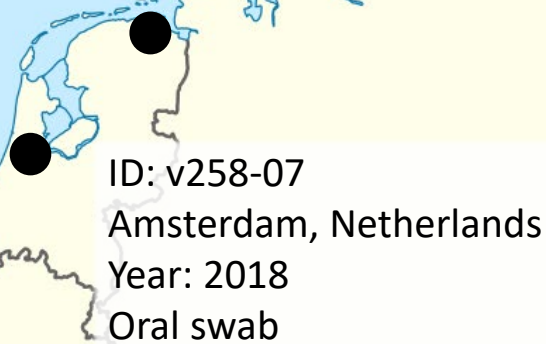

Supplement: Supplementary file 1 — Supplementary file1 Figure S1 Geographic map depicting T. depauwii isolate overview including clinical information. Figure adapted from //commons.wikimedia.org/wiki/File:North_Sea_location_map.svg and https://en.m.wikipedia.org/wiki/File:USA_Northeastern.png (PDF 216 KB) [file 15010_2024_2229_MOESM1_ESM.pdf]

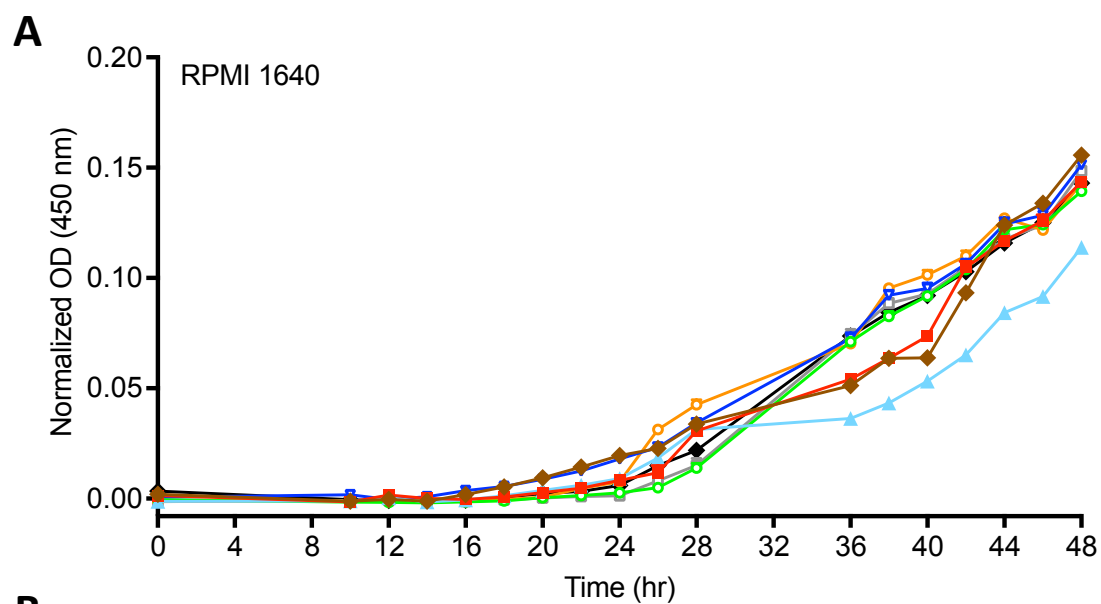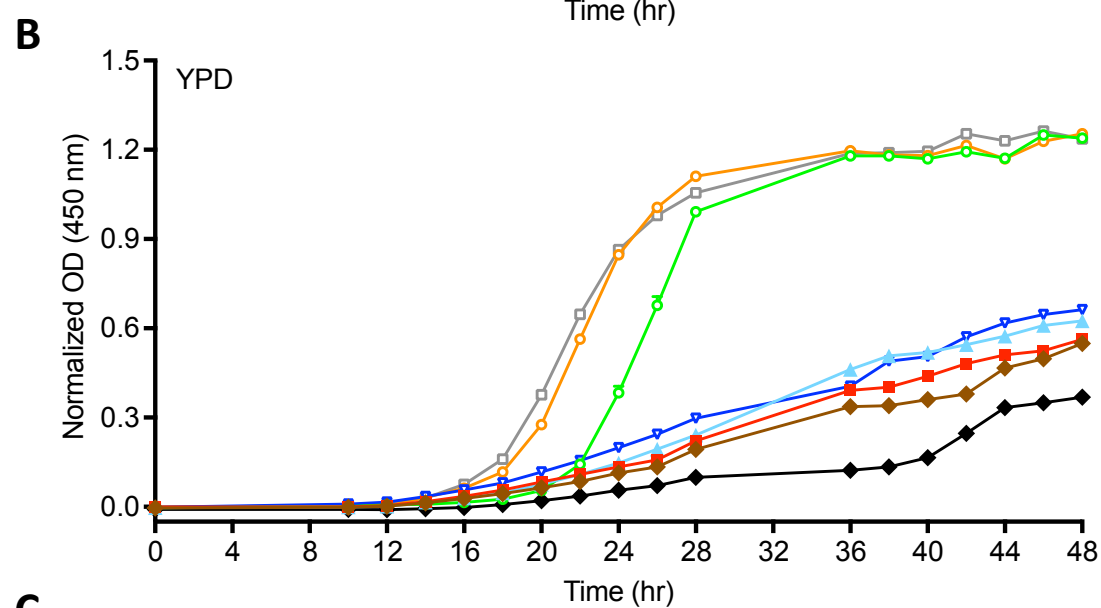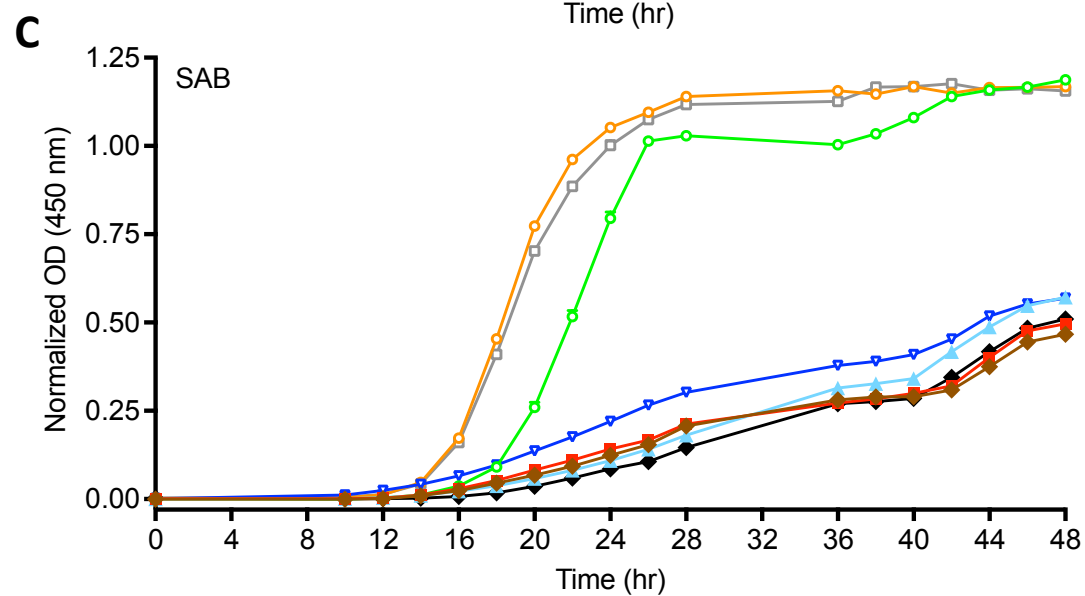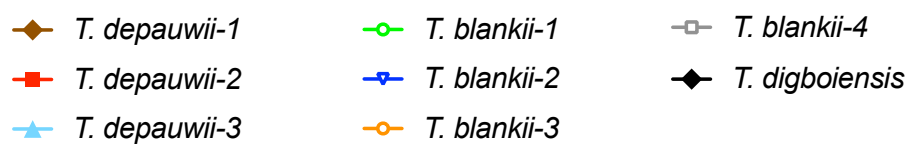

Supplement: Supplementary file 2 — Supplementary file2 Figure S2 Growth curves of T. depauwii (n = 3), T. blankii (n = 4) and T. digboiensis (n = 1) cultured in RPMI 1640 (0.2% glucose) medium (A), YPD (B) and SAB (C) broth at 35 °C. Strains were grown in eightfold and growth is expressed in average normalized OD values (PDF 129 KB) [file 15010_2024_2229_MOESM2_ESM.pdf]

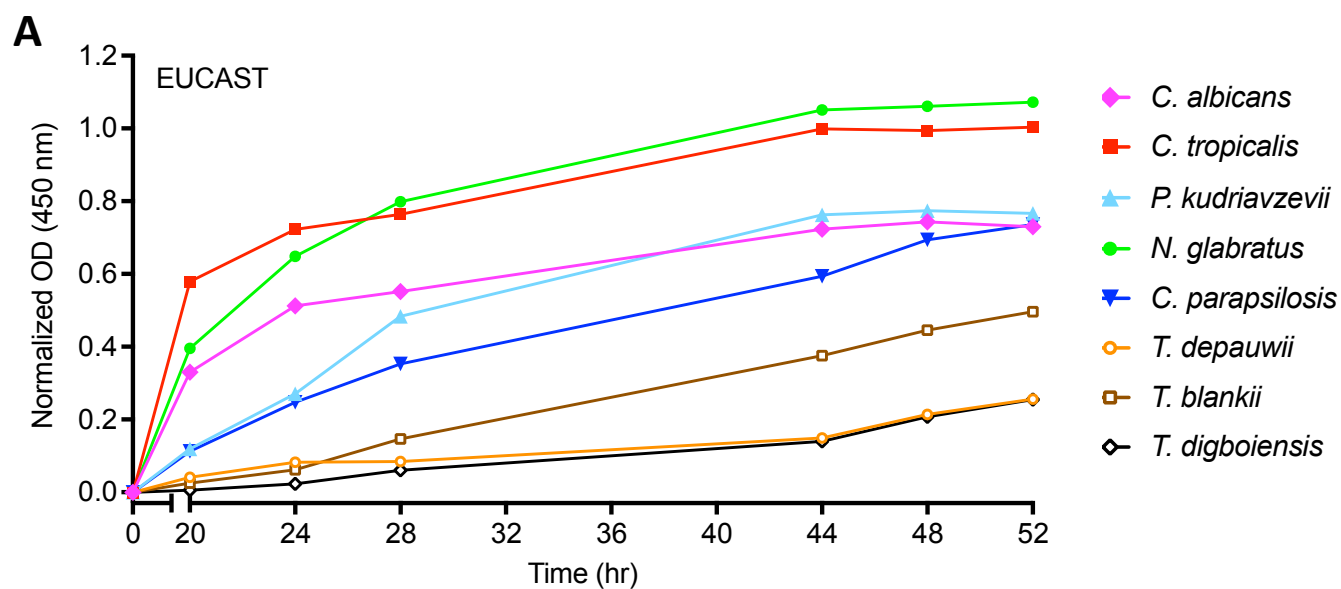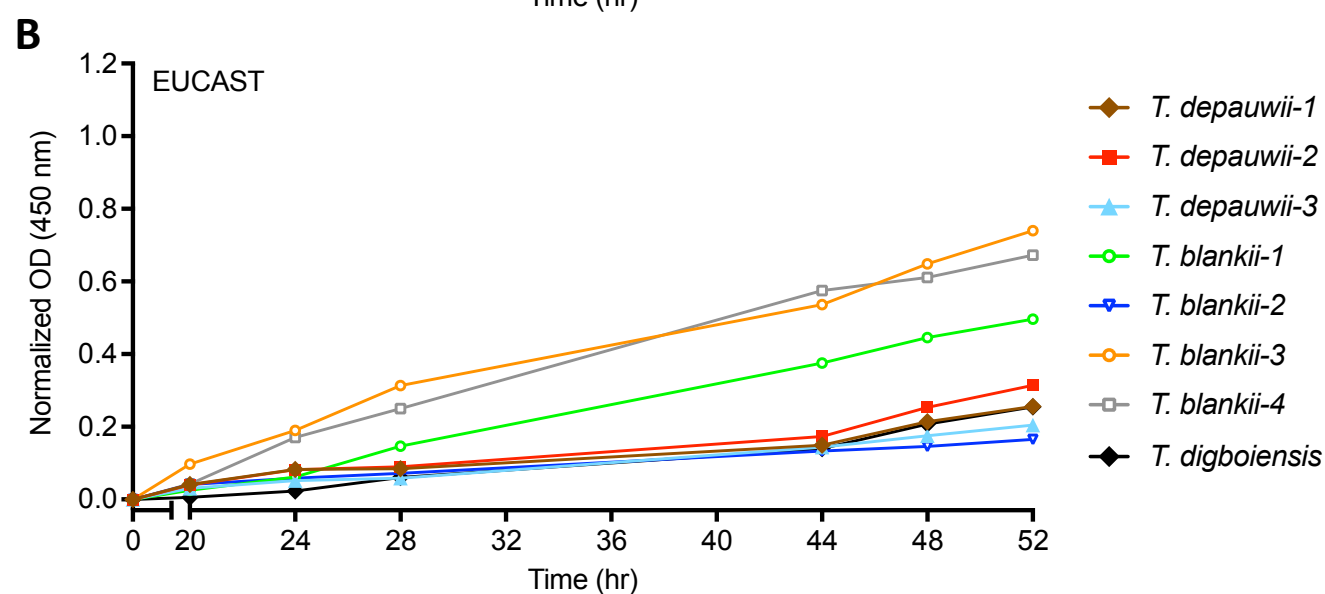

Supplement: Supplementary file 3 — Supplementary file3 Figure S3 Growth curves of Tardiomyces and common yeast species cultured in RPMI 1640 (2% glucose) medium at 35 °C. On top (A), one strain of each Tardiomyces species is displayed in addition to controls, and on bottom (B) solely Tardiomyces strains are shown. Strains were grown in eightfold and growth is expressed in average normalized OD values (PDF 75 KB) [file 15010_2024_2229_MOESM3_ESM.pdf]
