# Supplementary material for: The yeast genus Tardiomyces gen. nov. with one new species and two new combinations
Source: Infection. 2024 Apr 4;52(5):1799–812. doi: 10.1007/s15010-024-02229-6 (PMC11499460; doi:10.1007/s15010-024-02229-6)
Supplement: Supplementary file 4 — Supplementary file4 (DOCX 27 KB) [file 15010_2024_2229_MOESM4_ESM.docx]

**Table S1:** Isolate overview of characterized strains used in this study.

| **ID** | **Source** | **City, country** | **Year** |
| --- | --- | --- | --- |
| 1168/91 | Blood | Oslo, Norway | 1991 |
| NCPF8710 | Blood | London, UK | 2003 |
| NCPF8811 | Sputum | London, UK | 2005 |
| NCPF8830 | Unknown | UK | 2005 |
| NCPF13061 | Urine | Edinburgh, UK | 2013 |
| NCPF13062 | Blood | Eastbourne, UK | 2018 |
| NCPF13064 | Blood | Stoke, UK | 2018 |
| M.025-78 | Sputum | Groningen, The Netherlands | 2021 |
| V258-07 | Oral swab | Amsterdam, The Netherlands | 2018 |
| IDR21-363 | Blood | New York, USA | 2021 |
| Kw593/18 | Blood | Kuwait | 2018 |
| 76/2019 | Blood | São Paulo, Brazil | 2018 |
| 77/2019 | Sugar cane | Brazil | 2018 |
| 78/2019 | Sugar cane | Brazil | 2018 |
| 79/2019 | Sand | Rio de Janeiro, Brazil | 2019 |
| CBS 1898 | Mink organs | Canada | 1968 |
| CBS 2774 | Damp sulphite pump | Sweden | 1956 |
| CBS 6734 | Soil | Tokyo, Japan | 1987 |
| CBS 7205 | Horse uterus | New Zealand | 1987 |
| CBS 9800 | Tar-contaminated Soil | Digboi, India | 2005 |
| 963/P/16 | Blood | Delhi, India | 2016 |
| 1130/P/16 | Blood | Delhi, India | 2016 |
| 1168/P/16 | Blood | Delhi, India | 2016 |
| 1175/P/16 | Blood | Delhi, India | 2016 |
| 45/P/17 | Blood | Delhi, India | 2017 |
| 10-13-09-76 | Unknown | Amsterdam, Netherlands | Unknown |
| 10-13-10-05 | Blood | Lahore, Pakistan | 2020 |

**Table S2:** Overview of ITS sequences with accession numbers used in the current study.

| **Species** | **Strain** | **Source** | **City, country** | **Genbank accession #** |
| --- | --- | --- | --- | --- |
| *Candida albicans* | CBS 562 | Human skin | Uruguay | NR_125332.1 |
| *Crinitomyces ghanaensis* | CBS 8798 | Soil | Ghana | KY102101.1 |
| *Diddensiella caesifluorescens* | NCAIM Y.01956 | Rotten wood | Hungary | JF895510.1 |
| *Geotrichum candidum* | AUMC 8995 | Onychomycosis | Egypt | KU176111.1 |
| *Spencermartinsiella ligniputridi* | CBS 12585 | Rotten wood | Hungary | KY105462.1 |
| *Sugiyamella lignohabitans* | ATCC MYA-4663 | Beetle gut | Virginia, USA | HM208608.1 |
| *Tardiomyces blankii* | MUCL 29808 | Unknown | Unknown | NR_111312.1 |
| *Tardiomyces blankii* | C-9/336 | Blood | Bangladesh | OQ606980.1 |
| *Tardiomyces blankii* | IIC1M.1 | Unknown | Brazil | MF940143.1 |
| *Tardiomyces blankii* | 10-13-10-05 | Blood | Lahore, Pakistan | OR479767 |
| *Tardiomyces blankii* | NCPF8710 | Blood | London, UK | OR479768 |
| *Tardiomyces blankii* | NCPF8830 | Unknown | UK | OR479769 |
| *Tardiomyces blankii* | NCPF8811 | Sputum | London, UK | OR479770 |
| *Tardiomyces blankii* | 10-13-09-76 | Unknown | Amsterdam, The Netherlands | OR479771 |
| *Tardiomyces blankii* | 76_2019 | Blood | São Paulo, Brazil | OR479772 |
| *Tardiomyces blankii* | 77_2019 | Sugarcane | Brazil | OR479773 |
| *Tardiomyces blankii* | 78_2019 | Sugarcane | Brazil | OR479774 |
| *Tardiomyces blankii* | 79_2019 | Sand | Rio de Janeiro, Brazil | OR479775 |
| *Tardiomyces blankii* | Kw593/18 | Blood | Kuwait | OR479776 |
| *Tardiomyces blankii* | CBS 1898 | Mink organs | Canada | OR479777 |
| *Tardiomyces blankii* | CBS 2774 | Damp sulphite pump | Sweden | OR479778 |
| *Tardiomyces blankii* | CBS 6734 | Soil | Tokyo, Japan | OR479779 |
| *Tardiomyces blankii* | CBS 7205 | Horse uterus | New Zealand | OR479780 |
| *Tardiomyces blankii* | 963/P/16 | Blood | Delhi, India | OR479782 |
| *Tardiomyces blankii* | 1130/P/16 | Blood | Delhi, India | OR479783 |
| *Tardiomyces blankii* | 1168/P/16 | Blood | Delhi, India | OR479784 |
| *Tardiomyces blankii* | 1175/P/16 | Blood | Delhi, India | OR479785 |
| *Tardiomyces blankii* | 45/P/17 | Blood | Delhi, India | OR479786 |
| *Tardiomyces depauwii* | 1168/91 | Blood | Oslo, Norway | OR479792 |
| *Tardiomyces depauwii* | NCPF13061 | Urine | Edinburgh, UK | OR479787 |
| *Tardiomyces depauwii* | NCPF13062 | Blood | Eastbourne, UK | OR479789 |
| *Tardiomyces depauwii* | NCPF13064 | Blood | Stoke, UK | OR479788 |
| *Tardiomyces depauwii* | v258_07 | Oral swab | Amsterdam, The Netherlands | OR479790 |
| *Tardiomyces depauwii* | M.025-78 | Sputum | Groningen, The Netherlands | OR479791 |
| *Tardiomyces digboiensis* | CBS 9801 | Tar-contamined soil | India | KY102052.1 |
| *Tardiomyces digboiensis* | NB | Heap leaching plant | Zambia | KF659595.1 |
| *Tardiomyces digboiensis* | CBS 9800 | Tar-contamined soil | Digboi, India | OR479781 |

**Table S3:** Overview of genomes with corresponding order, strain, and accession number used in the phylogenomic analyses.

| **Order** | **Species** | **Strain** | **Genome Accession** |
| --- | --- | --- | --- |
| *Alaninales* | *Nakazawaea peltata* | JCM 9829 | GCA_001599355.1 |
| *Alaninales* | *Pachysolen tannophilus* | NRRL Y-2460 | GCA_001661245.1 |
| *Alloascoideales* | *Alloascoidea africana* | NRRL Y-6762-3 | GCA_030581615.1 |
| *Alloascoideales* | *Alloascoidea hylecoeti* | JCM 7604 | GCA_001600815.1 |
| *Ascoideales* | *Saccharomycopsis fibuligera* | KPH12 | GCA_001936155.1 |
| *Ascoideales* | *Saccharomycopsis schoenii* | CBS 7425 | GCA_010994365.1 |
| *Dipodascales* | *Tardiomyces blankii* | ABL | GCA_024734315.1 |
| *Dipodascales* | *Tardiomyces depauwii* | NCPF13064 | JAVHZA000000000 |
| *Dipodascales* | *Tardiomyces digboiensis* | CBS 9800 | GCA_030570855.1 |
| *Dipodascales* | *Crinitomyces ghanaensis* | NRRL YB-1486T | GCA_030581755.1 |
| *Dipodascales* | *Diddensiella santjacobensis* | NRRL Y-17667 | GCA_030574215.1 |
| *Dipodascales* | *Spencermartinsiella ligniputridi* | NRRL Y-48818 | GCA_030583385.1 |
| *Dipodascales* | *Sugiyamaella lignohabitans* | CBS 10342 | GCA_001640025.2 |
| *Dipodascales* | *Geotrichum candidum* | LMA-244 | GCA_013365045.1 |
| *Lipomycetales* | *Lipomyces mesembrius* | NRRL Y-27506 | GCA_003705845.2 |
| *Lipomycetales* | *Lipomyces oligophaga* | NRRL Y-17247 | GCA_003707545.2 |
| *Outgroup* | *Schizosaccharomyces pombe* | 972h- | GCA_000002945.2 |
| *Outgroup* | *Cryptococcus neoformans* | JEC21 | GCA_000091045.1 |
| *Phaffomycetales* | *Wickerhamomyces anomalus* | NRRL Y-366-8 | GCF_001661255.1 |
| *Phaffomycetales* | *Cyberlindnera jadinii* | NRRL Y-1542 | GCA_001661405.1 |
| *Pichiales* | *Komagataella phaffii* | GS115 | GCA_000027005.1 |
| *Pichiales* | *Ogataea parapolymorpha* | DL-1 | GCA_000187245.3 |
| *Pichiales* | *Pichia kudriavzevii* | CBS 573 | GCA_003054445.1 |
| *Saccharomycetales* | *Kluyveromyces marxianus* | DMKU3-1042 | GCA_001417885.1 |
| *Saccharomycetales* | *Saccharomyces cerevisiae* | S288C | GCA_000146045.2 |
| *Saccharomycetales* | *Nakaseomyces glabratus* | CBS 138 | GCA_000002545.2 |
| *Saccharomycodales* | *Hanseniaspora uvarum* | QTX-C10 | GCA_030180015.1 |
| *Saccharomycodales* | *Hanseniaspora vineae* | T02/19AF | GCA_000585475.3 |
| *Serinales* | *Candida albicans* | SC5314 | GCA_000182965.3 |
| *Serinales* | *Candida parapsilosis* | CDC317 | GCA_000182765.2 |
| *Serinales* | *Candida tropicalis* | MYA-3404 | GCA_000006335.3 |
| *Serinales* | *Candida dubliniensis* | CD36 | GCA_000026945.1 |
| *Serinales* | *Debaryomyces hansenii* | CBS767 | GCA_000006445.2 |
| *Serinales* | *Meyerozyma guilliermondii* | ATCC 6260 | GCA_000149425.1 |
| *Serinales* | *Candida auris* | B11220 | GCA_003013715.2 |
| *Serinales* | *Clavispora lusitaniae* | ATCC 42720 | GCA_000003835.1 |
| *Serinales* | *Candida intermedia* | CBS 141442 | GCA_900106115.1 |
| *Sporopachydermiales* | *Sporopachydermia lactativora* | NRRL Y-11591 | GCA_003705295.1 |
| *Sporopachydermiales* | *Sporopachydermia quercuum* | JCM 9486 | GCA_001599295.1 |
| *Trigonopsidales* | *Trigonopsis variabilis* | NRRL Y-1579 | GCA_003707065.2 |
| *Trigonopsidales* | *Tortispora caseinolytica* | NRRL Y-17796 | GCA_001661475.1 |

**Table S4:** Overview of substrates with variable assimilation patterns for *Tardiomyces blankii* strains.

| **Strain** | **Galactose** | **Methyl-α-D-glucoside** | **Cellobiose** | **Citrate** | **N-acetyl-Glucosamine** |
| --- | --- | --- | --- | --- | --- |
| NCFP8710 | + | + | + | - | + |
| NCPF8811 | + | - | + | - | - |
| NCPF8830 | + | + | + | - | + |
| Kw593/18 | + | + | + | - | + |
| 76/2019 | + | + | + | - | + |
| 77/2019 | + | + | + | - | + |
| 78/2019 | + | + | + | - | + |
| 79/2019 | + | - | - | - | - |
| CBS 1898 | + | + | + | - | + |
| CBS 2774 | + | + | + | + | - |
| CBS 6734 | - | - | + | - | - |
| CBS 7205 | + | + | + | - | + |
| 963/P/16 | + | + | + | - | + |
| 1130/P/16 | + | + | + | - | + |
| 1168/P/16 | + | + | + | - | + |
| 1175/P/16 | + | + | + | + | + |
| 45/P/17 | + | + | + | - | + |
| 10-13-09-76 | - | + | + | - | + |
| 10-13-10-05 | - | + | + | - | + |

Scoring system according to Kurtzman et al. [45], +: positive test, -: negative test.

**Table S5:** Biochemical profiles of *Tardiomyces depauwii, T. blankii, T. digboiensis,* according to API 20C AUX*.*

| **Substrate** | ***T. depauwii* (n=7)** | ***T. blankii* (n=19)** | ***T. digboiensis* (n=1)** |
| --- | --- | --- | --- |
| Glycerol | - | - | - |
| Glucose | + | + | + |
| L-arabinose | + | + | + |
| D-xylose | + | + | + |
| Xylitol | + | + | + |
| D-sorbitol | + | + | - |
| N-acetyl-glucosamine | + | +* | + |
| D-cellobiose | + | +* | + |
| Lactose | + | + | - |
| D-maltose | + | + | + |
| Sucrose | + | + | + |
| D-trehalose | + | + | - |
| D-melezitose | + | + | + |
| D-raffinose | - | - | + |
| 2-keto-gluconate | + | + | - |
| Methyl-α-glucopyranoside | + | +* | + |

Scoring system according to Kurtzman et al. [45], +: positive test, -: negative test. *assimilation results were variable between strains.

**Table S6:** In vitro AFST minimal inhibitory concentrations for *Tardiomyces depauwii* (n=7), *T. blankii* (n=4) and *T. digboiensis* (n=1) according to Sensititre YeastOne YO10 after 48 hours incubation. Minimal inhibitory concentrations in µg/mL. Growth inhibition MIC endpoints were determined regardless of the color.

| **Species** | **ID** | **AMB MIC** | **FLC MIC** | **ITR MIC** | **VOR MICC** | **POS MIC** | **AFG MIC** | **MFG MIC** | **CAS MIC** | **5FC MIC** |
| --- | --- | --- | --- | --- | --- | --- | --- | --- | --- | --- |
| *T. depauwii* | 1168/91 | 0.5 | ≥256 | 1 | 2 | 1 | 0.5 | 0.25 | 0.25 | ≤0.12 |
|  | M.025-78 | 0.5 | ≥256 | 0.5 | 8 | 1 | 0.5 | 0.06 | 0.12 | ≤0.12 |
|  | V258-07 | 0.5 | 128 | 0.5 | 4 | 0.5 | 0.12 | 0.12 | 0.12 | ≤0.12 |
|  | NCPF13061 | 2 | 128 | 0.5 | 4 | 1 | 1 | 0.25 | 0.25 | ≤0.12 |
|  | NCPF13062 | 1 | ≥256 | 2 | 8 | 1 | 4 | 0.25 | 0.25 | ≤0.12 |
|  | NCPF13064 | 2 | 128 | 1 | 2 | 1 | 2 | 0.5 | 0.5 | ≤0.12 |
|  | IDR21-363 | 1 | 256 | 1 | 8 | 1 | 2 | 0.5 | 0.5 | ≤0.12 |
| *T. blankii* | 79/2019 | 2 | 16 | 1 | 1 | 2 | 0.5 | 0.25 | 0.5 | ≤0.12 |
|  | Kw593/18 | ≤0.12 | 8 | 0.25 | 0.25 | 0.5 | 0.12 | 0.12 | 0.25 | ≤0.12 |
|  | 1130/P/16 | 2 | 16 | 0.5 | 1 | 1 | 1 | 0.12 | 0.25 | ≤0.12 |
|  | CBS 7205 | 1 | 16 | 0.5 | 0.25 | 1 | 1 | 0.12 | 0.5 | ≤0.12 |
| *T. digboiensis* | CBS 9800 | 1 | 8 | 0.125 | 0.25 | 0.125 | 2 | 2 | 4 | ≤0.12 |

MIC, minimal inhibitory concentration; AMB, amphotericin B; FLC, fluconazole; ITR, itraconazole; VOR, voriconazole; POS, posaconazole; AFG, anidulafungin; MFG, micafungin; CAS, caspofungin; 5FC, flucytosine.

**Table S7:** Antifungal susceptibility testing by E-test for *T. depauwii* strains. Minimal inhibitory concentrations in µg/mL.

| **ID** | **AMB MIC, 48 hrs** | **AMB MIC , 72 hrs** |
| --- | --- | --- |
| 1168/91 | 0.38 | 0.75 |
| M.025-78 | 0.38 | 0.5 |
| V258-07 | 0.75 | 0.75 |
| NCPF13061 | 1 | 1 |
| NCPF13062 | 0.38 | 0.5 |
| NCPF13064 | 1 | 1 |
| IDR21-363 | 0.75 | 1 |

AMB, amphotericin B; MIC
